# SlpB Protein Enhances the Probiotic Potential of *L. lactis* NCDO 2118 in Colitis Mice Model

**DOI:** 10.3389/fphar.2021.755825

**Published:** 2021-12-20

**Authors:** Giovanna A. Belo, Bárbara F. Cordeiro, Emiliano R. Oliveira, Marina P. Braga, Sara H. da Silva, Bruno G. Costa, Flaviano dos S. Martins, Gwénaël Jan, Yves Le Loir, Alfonso Gala-García, Enio Ferreira, Vasco Azevedo, Fillipe L. R. do Carmo

**Affiliations:** ^1^ Institute of Biological Sciences, Federal University of Minas Gerais (UFMG), Belo Horizonte, Brazil; ^2^ INRAE, STLO, Institut Agro, Agrocampus Ouest, Rennes, France; ^3^ School of Dentistry, Federal University of Bahia (UFBA), Salvador, Brazil

**Keywords:** SlpB, propionibacterium, colitis, *Lactococcus lactis*, inflammatory bowel disease

## Abstract

Bacteria used in the production of fermented food products have been investigated for their potential role as modulators of inflammation in gastrointestinal tract disorders such as inflammatory bowel diseases (IBD) that cause irreversible changes in the structure and function of gut tissues. Ulcerative colitis (UC) is the most prevalent IBD in the population of Western countries, and it is marked by symptoms such as weight loss, rectal bleeding, diarrhea, shortening of the colon, and destruction of the epithelial layer. The strain *Propionibacterium freudenreichii* CIRM-BIA 129 recently revealed promising immunomodulatory properties that greatly rely on surface-layer proteins (Slp), notably SlpB. We, thus, cloned the sequence encoding the SlpB protein into the pXIES-SEC expression and secretion vector, and expressed the propionibacterial protein in the lactic acid bacterium *Lactococcus lactis* NCDO 2118. The probiotic potential of *L. lactis* NCDO 2118 harboring pXIES-SEC:*slpB* (*L. lactis*-SlpB) was evaluated in a UC-mice model induced by Dextran Sulfate Sodium (DSS). During colitis induction, mice receiving *L. lactis*-SlpB exhibited reduced severity of colitis, with lower weight loss, lower disease activity index, limited shortening of the colon length, and reduced histopathological score, with significant differences, compared with the DSS group and the group treated with *L. lactis* NCDO 2118 wild-type strain. Moreover, *L. lactis*-SlpB administration increased the expression of genes encoding tight junction proteins *zo-1*, *cln*-1, *cln-5*, *ocln*, and *muc-2* in the colon, increased IL-10 and TGF-β, and decreased IL-17, TNF-α, and IL-12 cytokines in the colon. Therefore, this work demonstrates that SlpB recombinant protein is able to increase the probiotic potential of the *L. lactis* strain to alleviate DSS-induced colitis in mice. This opens perspectives for the development of new approaches to enhance the probiotic potential of strains by the addition of SlpB protein.

## Introduction

*Propionibacterium freudenreichii* (*Pf*) is a dairy propionic acid bacterium (PAB) that has gained prominence as a potential probiotic, after studies have shown primitive characteristics, such as the production of short-chain fatty acids and conjugated linoleic acid, in addition to producing vitamin 12 at an industrial scale (Thierry et al., 2011; Deptula et al., 2017). *Pf* has been listed in the qualified presumption of safety (QPS) list by the European food safety authority and has a GRAS (Generally Recognized As Safe) status for its use in cheese. The immunomodulatory properties of some *Pf* strains have already been clearly demonstrated in inflammatory bowel disease (IBD) mice models (Foligne et al., 2010; Carvalho et al., 2017; Ma et al., 2020; Rabah et al., 2020) and in the mucositis model (Cordeiro et al., 2018; Do Carmo et al., 2020). The probiotic properties of *Pf* are directly linked to the presence of surface proteins, the S-layer proteins (Slp), as shown by the study by Do Carmo et al. (2017) and Do Carmo et al. (2018). Mutation of the gene encoding SlpB, a surface protein present at the surface of the probiotic strain *P. freudenreichii* CIRM-BIA 129 (*Pf* 129), drastically alters its immunomodulatory effects *in vitro* and *in vivo*, its adhesion to HT-29, its physicochemical properties, its ability to survive stress, and its surface and whole-cell proteome. Moreover, the purified *Pf* 129 SlpB protein was able to increase IL-10 gene expression in HT-29 cells. Furthermore, it is very important to know whether the immunomodulatory effects of the *Pf* 129 SlpB protein can be observed in other organisms, such as lactic acid bacteria.

Probiotic potential of the lactic acid bacterium (LAB) *Lactococcus lactis* has widely been explored. Notably, recent studies on the *L. lactis* subsp. *lactis* strain NCDO 2118 pointed out its potential to control intestinal inflammation in a mouse model (Carvalho et al., 2017). Precisely, *L. lactis* NCDO 2118 is amenable to transformation, and it has been used for the production and secretion of heterologous proteins in a *L. lactis* species that reportedly secretes a small number of homologous proteins (Nouaille et al., 2003). Miyoshi and collaborators (2004) developed a versatile plasmidic expression system inducible by xylose (xylose-inducible expression system—XIES). XIES plasmid (pXIES) can address the recombinant protein to the cytoplasm (p*XIES*-*CYT*) or to the extracellular medium (p*XIES*-*SEC*) (Miyoshi et al., 2004). Gomes-Santos et al. (2017) explored the potential of *L. lactis* strain NCDO 2118 secreting the *Mycobacterium leprae* heat-shock protein HSP65 (p*XIES*-*SEC*:*hsp*65) and obtained promising results in mitigating experimental colitis in mice model.

Probiotics, such as *P. freudenreichii* CIRM-BIA 129 and *L. lactis* NCDO 2118, have been tested as adjuvants in the treatment of colitis in animal models (Gomes-Santos et al., 2017; Ma et al., 2020; Rabah et al., 2020; Cordeiro et al., 2021). IBDs induce pathological signs and symptoms such as weight loss, rectal bleeding, diarrhea, shortening of the colon, and destruction of the epithelial layer. In the colon mucosa of patients affected by ulcerative colitis (UC), the presence of an inflammatory infiltrate composed of neutrophils and eosinophils are described. Furthermore, destruction of the epithelial barrier and the mucin layer leads to the exposure to antigens or pathobionts present in the intestinal lumen, exacerbating the pro-inflammatory response (Kushkevych and Monika, 2021). IBD etiology is being explored to unravel the mechanisms responsible for this pathology. Beyond the evidence of genetic susceptibility, the intestinal microbiota alterations (or dysbiosis), causing an exacerbated immune response in the host, can affect and aggravate IBD symptoms. An experimental approach proposed for the study of IBDs is a mice model of colitis induced by Dextran Sulfate Sodium (DSS). DSS-induced colitis model is able to mimic and reproduce IBD pathology routinely observed in human UC, body weight reduction, diarrhea, bloody feces, decreased colon length, mucosal injury, impaired mucus epithelial barrier function, and proinflammatory immune response (Wirtz et al., 2017).

In this work, we explore the therapeutic role of *Pf* 129 SlpB protein in the modulation of intestinal inflammations induced by chemical substances. In this aim, we use the *L. lactis* NCDO2118 harboring pXYSEC:*slpB*, to evaluate its effects in the DSS-colitis mice model.

## Materials and Methods

### Strains and Cloning Procedure

*Lactococcus lactis* NCDO 2118 wild-type (*L. lactis* WT) strain was grown at 30°C in M17 medium (Difco) containing 0.5% glucose (GM17), without agitation, or in the same medium solidified with 1.5% agar for 18 h. The nucleotide sequence encoding the SlpB surface protein from *Propionibacterium freudenreichii* CIRM-BIA 129 (*Pf* 129) was obtained from the database of the National Center for Biotechnology Information (NCBI), deposited under accession number CDP48273.1 (CDS 5503..7173). The sequence was optimized for expression in *Lactococcus lactis* NCDO2118 bacteria in the OptimumGeneTM program (GenScript Corporation) and synthesized by GenScript Corporation (Piscataway, NJ, USA) and cloned into pUC57 vector. The optimized SlpB protein sequence was synthesized with the restriction sites *Nsi*I-3′ and *Eco*RI-5 to cloning into the plasmid pXYSEC (chloramphenicol resistance). The NsiI/EcoRI digested and purified SlpB ORF and pXY:SEC fragments were ligated by T4 DNA ligase (Invitrogen) to obtain the pXYSEC*:slpB* plasmid, which was established by transformation in *E. coli* Top10 and selected with 10 μg/ml of chloramphenicol (Cm) in Luria Bertani Agar (Miyoshi et al., 2004). Routinely, the *primers* SlpB-Forward 5′-GAT​CCC​CCG​TCT​GAA​CGA​ACT​T-3′ and SlpB-Reverse 5′-CGA​CAT​CAT​TGA​ACA​TGC​TGA​AGA​GC-3′ were used for plasmid construction verification by PCR and agarose gel, and also for sequencing (PCR product size: 1,816 bp). Then, the optimized gene sequence for SlpB was subcloned into the pXYSEC plasmid and competent *L. lactis* NCDO 2118 bacteria was transformed by electroporation as previously described by Langella et al. (1993), and grown at 30°C in M17 medium (Difco) containing 0.5% glucose (GM17) without agitation containing 10 μg/ml of chloramphenicol. To confirm the final construction of pXYSEC:*slpB*, a DNA sequencing was performed by fluorochrome-labeled dideoxynucleotides method (BigDye Terminator v3.1 Cycle Sequencing, Applied Biosystems, USA). Recombinant *L. lactis* NCDO2118 strain was grown in Difco M17 broth, supplemented with either 0.5% glucose (GM17) or 1% xylose (XM17) and chloramphenicol (10 μg/ml) at 30°C without agitation. On the first day, single colonies of recombinant *L. lactis* NCDO2118 harboring pXYSEC:*slpB* (*L. lactis-*SlpB) were cultured in 5 ml of GM17. On the second day, the overnight culture was diluted 1:10,000 in XM17 to induce the expression of the *slpB* gene ORF. Proteins sample preparation from *L. lactis* wild type and recombinant *L. lactis-*SlpB cultures was performed as previously described (Miyoshi et al., 2004). To verify protein production, a Sodium dodecyl sulfate-polyacrylamide gel electrophoresis (SDS–PAGE) and Western blotting was done as previously described by Do Carmo et al., 2017.

### Animals

Conventional female C57BL/6 mice of 8 weeks of age, obtained at the Universidade Federal de Minas Gerais (UFMG, Belo Horizonte, Brazil), were used in this work. They were housed in plastic cages in a room with controlled temperature (18°C–23°C), light cycle 14 h light/10 h dark, relative humidity (40%–60%), and *ad-libitum* access to food and water. All experimental procedures realized in this work were approved by the Ethics Committee on Animal Experimentation of the Universidade Federal de Minas Gerais (CEUA-UFMG, Brazil) by the protocol no. 148/2020.

### Experimental Design and Dextran Sulfate Sodium-Induced Colitis

*L. lactis* NCDO2118 WT (*L. lactis*) and *L. lactis* NCDO2118 pXYSEC: *slpB* (*L. lactis*-SlpB) strains were prepared daily for the animals, using intragastric gavage as a form of administration. Both strains were grown in M17 medium (Difco) added with glucose (0.5%), for the wild-type strain, and xylose (1%) + 10 μg/ml of Cm, for the *L. lactis*-SlpB strain. Bacteria cultures (*L. lactis* and *L. lactis*-SlpB), were incubated for 24 h at 30°C, and 1 ml of each culture was centrifuged at 3,500 rpm for 10 min and washed with PBS pH 7.4 twice to remove the antibiotic. Thus, each bacterial pellet corresponds to a daily dose with 5 × 10^9^ CFU/per dose of bacteria, which was then resuspended in 100 µl of PBS pH 7.4.

The mice were divided randomly into four main groups, each containing six animals per group (Supplementary Figure S1). Group 1 represented a healthy control group that received only water for drinking (control group). The mice from groups 2–4 (experimental groups) received DSS (36–50 kDa, MP Biomedicals, CAT 260110, LOT Q5756), as the only drinking source, prepared to a concentration of 1.7% in filtered drinking water and provided to the animals daily, during 7 days, according to the acute colitis model previously described (Wirtz et al., 2017). Animals from group 2 received only DSS solution (group DSS) and no treatment. Mice from groups 3 and 4 received the bacteria dose during the 7 days by gavage (all experimental days), together with DSS in drinking water. Precisely, mice from group 3 received intragastric doses (100 µl containing 5 × 10^9^ CFU) of *L. lactis* NCDO 2118 WT (group DSS + NCDO 2118 WT), and animals in group 4 received (100 µl containing 5 × 10^9^ CFU) of *L. lactis* NCDO 2118 pXYSEC:*slpB* (group DSS + NCDO2118 pXYSEC:*slpB*). The mice were euthanized on the seventh day. All *in vivo* experiments were done in biological triplicate.

### Assessment of Colitis Severity

During all experimental days, the water, food intake, and mice body weight were recorded daily. On the last experimental day, the disease activity index (DAI) was determined, as described by Cooper et al. (1993), attributing a score of the three major colitis clinical signs: weight loss, intensity of diarrhea, and presence of rectal bleeding.

A longitudinal abdominal incision was performed in all mice to access the intestine and colon, and then to be used in future analyses. The colon length of each mouse (measured from the cecum to rectum) were used to indicate the mean of each experimental group (cm). The colon distal part was collected, washed with PBS, and stored in segment rolls for histomorphological analysis. These rolls were immersed in formaldehyde solution (4%, v/v) for tissue fixation and, after that, they were embedded in paraffin. A section (4 µm) was placed on a glass slide and stained with Hematoxylin-Eosin (HE) (Marchal Bressenot et al., 2015). The sections were photographed (×20 magnification objective) using a digital camera (Spot Insight Color) coupled to an optical microscope (Olympus, BX-41, Japan). The histological inflammation score was determined by a pathologist using the score previously described by Wirtz et al. (2017). Consider: tissue damage (0: none; 1: isolated focal epithelial damage; 2: mucosal erosions and ulcerations; 3: extensive damage deep into the bowel wall) and lamina propria inflammatory cell infiltration (0: infrequent; 1: increased, some neutrophils; 2: the submucosal presence of inflammatory cell clusters; 3: transmural cell infiltrations). The total score ranging from 0 (no changes) to 6 (widespread cellular infiltrations and extensive tissue damage) were obtained by the sum of these two sub-scores (tissue damage and lamina propria inflammatory cell infiltration). Other cuts of the paraffinized colon samples were produced and stained by the periodic acid-Schiff (PAS) (Prisciandaro et al., 2011) in order to count the mucus-producing goblet cells. Ten random field images from each sample were made using the ×40 objective, and then, using ImageJ software (version 1.8.0) the intact goblet cells were counted. The total number of goblet cells was expressed as the number of cells per high-power field (HPF) (×40, 108.2 µm^2^).

### Colonic Activity of Myeloperoxidase and the Eosinophil Peroxidase

Neutrophil infiltration levels in the colon tissue were assessed by measurement of myeloperoxidase activity (MPO), as previously described by Porto et al., 2019. For MPO quantification, a piece of colon tissue (100 mg) was homogenized proportionally in 1.9 ml/100 mg of PBS and centrifuged at 10,000 × *g* for 10 min. The pellet formed was lysed and centrifuged again. The pellet formed was resuspended proportionally in 1.9 ml/100 mg of 0.5% HTAB (hexadecyltrimethylammonium bromide) diluted in PBS. The suspension was submitted to freeze–thaw cycle (3x) using liquid nitrogen and then, centrifuged at 12,000 × *g* at 4°C, for 10 min. In order to perform the enzymatic assay, we added an equal amount of substrate (1.5 mM L^−1^ of o-phenylenediamine and 6.6 mM L^−1^ of H_2_O_2_ in 0.075 mM L^−1^ of Tris–HCl pH 8.0) to the supernatant. To stop the enzymatic reaction, 50 μl of 1 M H_2_SO_4_ was added. The absorbance was read in a spectrophotometer (Spectramax M3, Molecular Devices, LLC, Sunnyvale, CA, USA), at 492 nm.

The extent of eosinophil infiltration into the tissues was assessed by measuring eosinophil peroxidase (EPO) activity, as previously described by Vieira et al. (2009). For EPO quantification, a piece of colon tissue (100 mg) was homogenized proportionally in 1.9 ml/100 mg of PBS and centrifuged at 10,000 × *g* for 10 min 4°C. The precipitate was subjected to hypotonic lysis, where 0.9 ml of a solution containing 0.2% NaCl was added prior to the addition of an equal volume of solution containing 1.6% NaCl and 5% glucose. The samples were again homogenized and centrifuged (10,000 × *g*, at 4°C, for 10 min). The supernatant was discarded, and the pellet was resuspended in 1.9 ml of 0.5% HTAB (hexadecyltrimethylammonium bromide) diluted in PBS. After three cycles of freeze–thaw in liquid nitrogen, the samples were centrifuged at 4°C, 10,000 g for 10 min. To test the enzyme activity, the obtained supernatant was mixed with a substrate (1:1) containing 1.5 mmol/L of o-phenylenediamine, 6.6 mmol/L of H_2_O_2_, and 0.075 mmol/L of Tris-HCl pH 8. After 30 min the reaction was stopped with 50 μl of 1 M H_2_SO_4_. The absorption was measured in a spectrophotometer (Spectramax M3, Molecular Devices, LLC, Sunnyvale, CA, United States) at 492 nm.

### Measurement of Secretory Immunoglobulin A

The secretory immunoglobulin A (sIgA) of the intestinal lavage was determined by ELISA, according to Cordeiro et al., 2021. For the quantification of the samples was used a 96 well-plates (Nunc-Immuno Plates, MaxiSorp) coated with anti-IgA antibodies (Southern Biotechnology, Birmingham, AL, United States) and incubated overnight. After the incubation, the plates were washed in saline-Tween (saline with 0.05% of Tween-20—SIGMA Chemical Co.) and blocked with 200 µl of PBS-casein (0.05%) for 1 h, at room temperature. After that, the intestinal lavage contents were added, and the plate was serially diluted (1:100) and incubated at room temperature for 1 h. Plates were washed with saline-Tween and then, biotin-conjugated anti-mouse IgA antibodies were added (Southern Biotechnology) (1: 10,000 in PBS-casein). Plates were incubated for 1 h at 37°C and then, biotinylated monoclonal antibodies anti-IgA (BD Bioscience) were added and incubated for 1 h at room temperature. Following this, peroxidase-labeled streptavidin (Southern Biotechnology) was added. Plates were washed in saline-Tween and incubated again with 100 µl of orthophenylenediamine (OPD) (Sigma, St. Louis, MO, USA) and H_2_O_2_ (0.04%), for 1 h, at room temperature. To stop the reaction, 20 µl/well of 2N H_2_SO_4_ was added. Absorbance reading was performed on Bio-Rad Model 450 Microplate Reader, at 492 nm. The results of total sIgA were measured, according to the standard curve, in a concentration of sIgA (ng) per ml of intestinal fluid.

### Colonic Gene Expression Analysis

In order to obtain the quantitative gene expression in colon fragments, the methodology was carried out according to Do Carmo et al. (2020). Fragments of 1 cm of the colon were collected. Total RNA was isolated using PureLink RNA Mini Kit (Thermo Fisher Scientific) according to the protocol of the manufacturer. Afterward, DNase I (Invitrogen; Waltham, MA, USA) was used to digest residual genomic DNA of samples, and then Turbo DNA-free Kit (Ambion; Austin, TX, USA) was used for DNA removal following the protocol of the manufacturer. RNA quality was checked by agarose gel and NanoDrop^®^ ND-1000 (260/230 ratio). To obtain the samples cDNA the High-Capacity cDNA Reverse Transcription kit (Applied Biosystems; Foster City, CA, United States) was used. Quantitative PCR (qPCR) was determined using iTaq universal SYBR green supermix (Biorad; Hercules, CA, United States) and gene specific primers (Table 1), for Mucin 2 (*muc-2*), Zonula occludens 1 (*zo-1*), zonula occludens 2 (*zo-2*), Claudin-1 (*cln-1*), Claudin-5 (*cln-5*), Occludin (*ocln*), inducible nitric oxide synthase (*inos*), peroxisome proliferator-activated receptor-gamma (pparg), and cytokine genes for interleukin-10 (*il-10*), *il-17*, as well as housekeeping genes encoding β-actin (*actβ*) and GAPDH (*gapdh*). The amplification cycles were performed as described: 95°C for 30 s, and 40 cycles of 95°C for 15 s and 60°C for 30 s on ABI PRISM 7900HT Sequence Detection System (Applied Biosystems). Results were expressed as a fold-change of expression levels, using the mean and standard deviations of target expression (2^−ΔΔCt^).

**TABLE 1:** Primer list for RT-quantitative PCR (qPCR).

Gene	Primer	Sequence (5′ → 3′)	References
*actβ*	Forward	TGG​CTG​GGT​GTT​GAA​GGT​CT	Do Carmo et al. (2020)
	Reverse	AGC​ACG​GCA​TCG​TCA​CCA​ACT	
*gapdh*	Forward	CAA​CGA​CCA​CTT​TGT​CAA​GC	Do Carmo et al. (2020)
	Reverse	TTC​CTC​TTG​TGC​TCT​TGC​TG	
*muc2*	Forward	CAG​CAC​CGA​TTG​CTG​AGT​TG	Do Carmo et al. (2020)
	Reverse	GCT​GGT​CAT​CTC​AAT​GGC​AG	
*zo1*	Forward	GAA​TGA​TGG​TTG​GTA​TGG​TGC​G	Do Carmo et al. (2020)
	Reverse	TCA​GAA​GTG​TGT​CTA​CTG​TCC​G	
*zo2*	Forward	GGA​GAC​CAG​ATT​CTG​AAG​GTG​AAC​ACA	Rabah et al. (2020)
	Reverse	CCT​TTG​GGG​ATT​TCT​AGC​AGG​TAG​AGG​AC	
*cld-1*	Forward	CTG​GAA​GAT​GAT​GAG​GTG​CAG​AA	Rabah et al. (2020)
	Reverse	CTA​ATG​TCG​CCA​GAC​CTG​AA	
*cld-5*	Forward	ACGGGAGGAGCGCTTTAC	Pfeiffer et al. (2011)
	Reverse	GTTGGCGAACCAGCAGAG	
*ocln*	Forward	GGA​CCC​TGA​CCA​CTA​TGA​AAC​AGA​CTA	Rabah et al. (2020)
	Reverse	TAG​GTG​GAT​ATT​CCC​TGA​CCC​AGT​C	
*inos*	Forward	CAG​CTG​GGC​TGT​ACA​AAC​CTT	Rabah et al. (2020)
	Reverse	CAT​TGG​AAG​TGA​AGC​GTT​TCG	
*pparg*	Forward	CAG​GCT​TCC​ACT​ATG​GAG​TTC	PlÉ et al. (2016)
	Reverse	GGC​AGT​TAA​GAT​CAC​ACC​TAT​CA	
*il10*	Forward	AAA​GAA​GGC​ATG​CAC​AGC​TC	Do Carmo et al. (2020)
	Reverse	AAG​CAT​GTT​AGG​CAG​GTT​GC	
*il17a*	Forward	GCT​CCA​GAA​GGC​CCT​CAG​A	Rabah et al. (2020)
	Reverse	AGC​TTT​CCC​TCC​GCA​TTG​A	

Note. *muc 2*, Mucin 2; *zo1*, *zonula* Occludes 1; *zo2*, *zonula* Occludes 2; *cln-1*, Claudin-1; *cln-5*, Claudin-5; *ocln*, Occludin; *inos*, inducible nitric oxide synthase; *pparg*, peroxisome proliferator-activated receptor-gamma; *il10*, interleukin-10.,

### Cytokine Quantification by Enzyme-Linked Immunosorbent Assay

For the quantification of cytokines, the samples were weighed, and 50 mg of colon tissue was homogenized in 1 ml of PBS solution containing Tween-20 (0.05%) (Sigma-Aldrich, St. Louis, MO, USA), Phenylmethylsulfonyl fluoride (PMSF) 0.1 mM (Sigma-Aldrich, St. Louis, MO, USA), 0.1 mM benzethonium chloride (Sigma-Aldrich, St. Louis, MO, USA), 10 mM EDTA (Synth, São Paulo, São Paulo, Brazil), and aprotinin A 20 KIU (Sigma-Aldrich, St. Louis, MO, USA). The homogenized samples were then centrifuged at 3,000 × *g* for 10 min at 4°C, and the supernatants were collected to perform the enzyme-linked immunosorbent assay (ELISA). Plates were coated with purified monoclonal antibodies reactive with cytokines IL-1β, IL-10, IL-12, p70, IL-17, TGFβ1, and TNF-α (B&D Systems, Inc., USA), overnight at 4°C. Then, plate wells were washed, supernatants were added, and the plates were again incubated overnight at 4°C. On the third day, biotinylated monoclonal antibodies against cytokines (R&D Systems, Inc., USA) were added to the plates and incubated for 2 h at room temperature. Color was developed at room temperature with 100 μl/well of orthophenylenediamine (1 mg/m) and 0.04% (v/v) H_2_O_2_ substrate in sodium citrate buffer. The reaction was stopped by the addition of 20 μl/well of 2N H_2_SO_4_. The absorbance was measured at 492 nm using a Microplate Reader Model 680 (BIO-RAD).

### Statistical Analyses

Data were analyzed using one-way ANOVA followed by Tukey’s post-test and performed in GraphPad Prism version 9.1 for Windows (GraphPad Software, San Diego, CA, USA). The experimental assays were performed in triplicate, and the results were expressed as mean ± standard deviation. Asterisks demonstrated in all figures represent the significant differences between the experimental groups and were indicated as follows: *p< 0.05; **p < 0.01; ***p < 0.001; ****p < 0.0001.

## Results

### *Lactococcus lactis-*Surface-Layer Protein B Reduces Weight Loss in Dextran Sulfate Sodium-Induced Colitis

**FIGURE 1:**
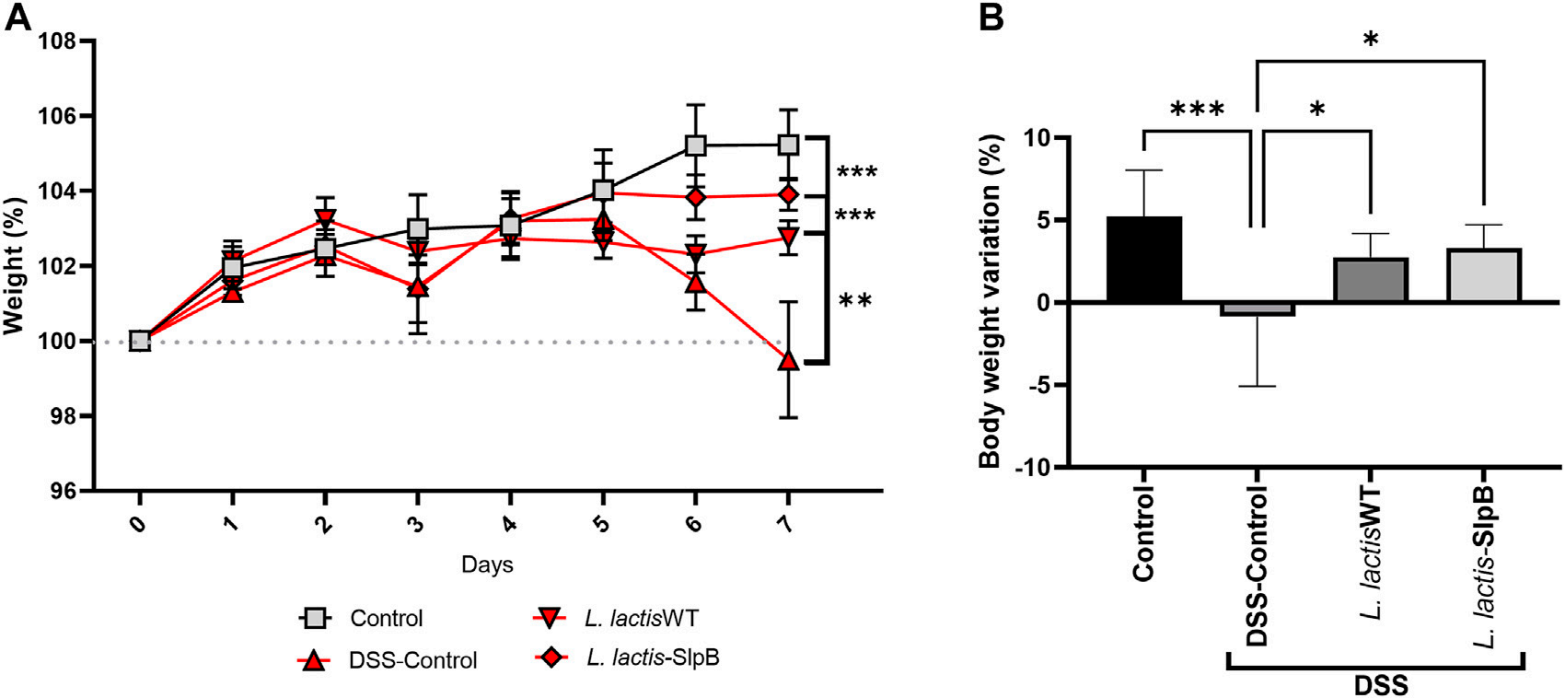
*Lactococcus lactis*-Surface-layer protein (Slp)B is able to control weight loss in Dextran Sulfate Sodium (DSS)-induced colitis. Time-course of body weight during the seven experimental days **(A)** and weight loss **(B)** are shown. The two-way ANOVA **(A)**, one-way ANOVA **(B)**, and Tukey’s *post*-*hoc* tests were used for the multiple comparisons (The data represent the mean ± SD of 12 mice per group). Asterisks represent statistically significant differences as follows: ∗*p* < 0.05, ∗∗*p* < 0.01, ∗∗∗*p* < 0.001, ∗∗∗∗*p* < 0.0001.

Expression of the *P. freudenreichii* SlpB protein by *L. lactis* NCDO 2118 was first verified by Western blotting (Supplementary Figure S2). We then investigated the ability of such expression to enhance the probiotic properties of the *L. lactis* in the context of DSS-colitis. First, the liquid and food consumption, as well as the weight of the animals, were monitored during the seven experimental days. In the DSS group, significant -weight loss was observed (*p* < 0.001 and *p* < 0.0001, respectively) on the sixth and seventh days (Figures 1A, B). However, at the end of the 7 days, both treatments with *L. lactis-*SlpB and the control group *L. lactis* WT were able to limit such weight loss of the animals (*p* < 0.001 and *p* < 0.01, respectively), when compared with the DSS group. Concerning liquid and food intake, no significant change was observed between experimental groups (Supplementary Figure S3).

### *Lactococcus lactis*-Surface-Layer Protein B Alleviates Clinical and Macroscopic Symptoms in Dextran Sulfate Sodium-Colitis Mice Model

**FIGURE 2:**
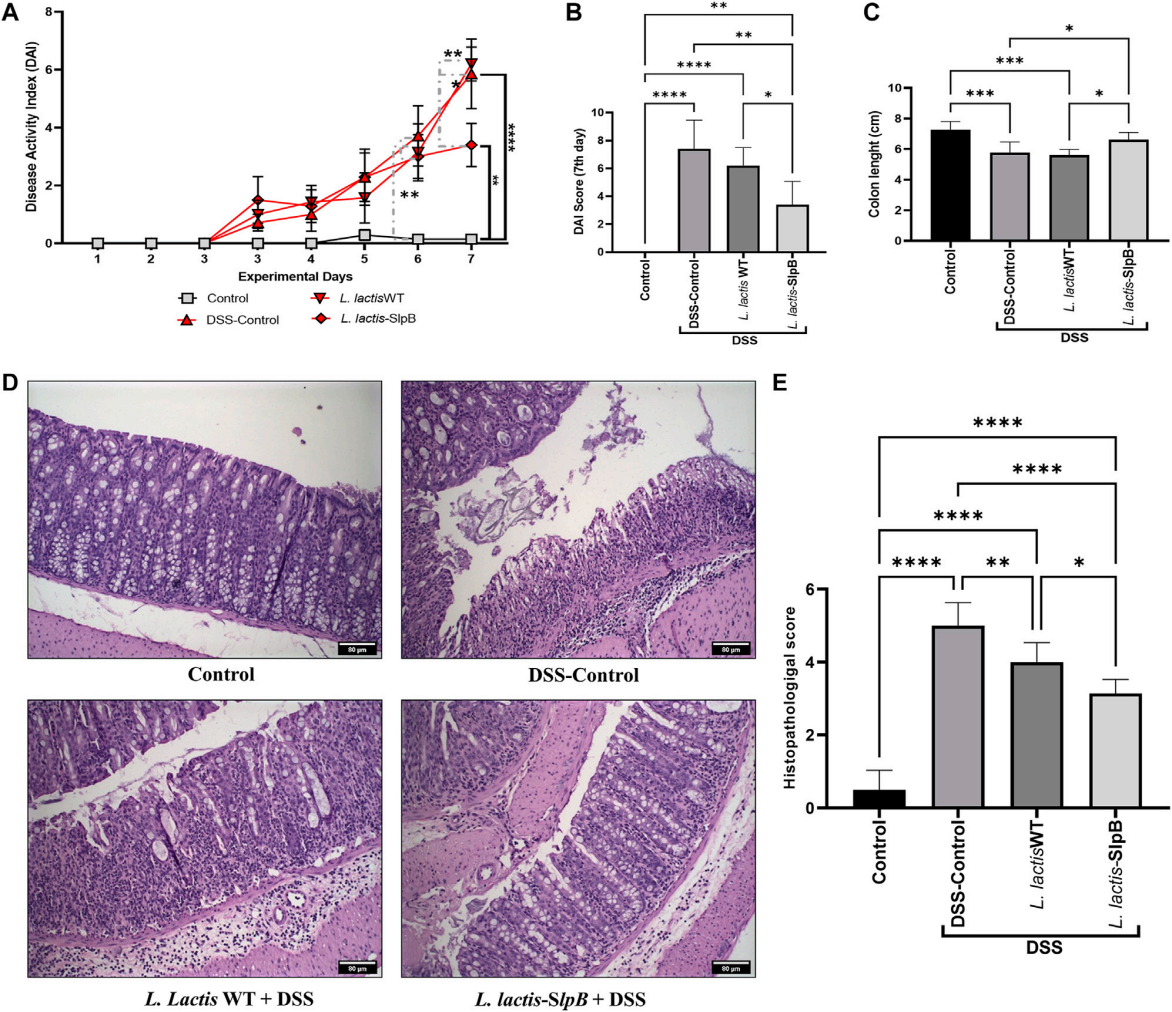
*L lactis*-SlpB alleviates clinical symptoms in DSS-colitis mice model and reduces colon mucosal damage. Disease activity index over the seven experimental days **(A)**, at the last day **(B)**, Colon length analysis **(C)**, Micrograph images of the histopathological analysis of the colon tissue **(D)** and analysis of the histopathological score **(E)** are shown. The slides were stained in hematoxylin and eosin (H&E) and analyzed under ×20 magnification. The two-way ANOVA **(A)**, one-way ANOVA **(B)**, and Tukey’s *post*-*hoc* tests were used for the multiple comparisons (The data represent the mean ± SD of 12 mice per group). Asterisks represent statistically significant differences as follows: ∗*p* < 0.05, ∗∗*p* < 0.01, ∗∗∗*p* < 0.001, ∗∗∗∗*p* < 0.0001.

Regarding disease activity index (DAI) analysis (Figures 2A, B), as expected, the DSS significantly increased the score (5.85 ± 3.18) in the disease control group (DSS Control), when compared with the healthy group (Control) on the sixth and seventh days (*p* < 0.05 and *p* < 0.0001, respectively). At the end of 7 days, *L. lactis-*SlpB administration was shown to mitigate the signs of clinical colitis, based on DAI score (3.40 ± 1.67), when compared with the DSS control (*p* < 0.01). Treatment with *L. lactis* WT strain failed, by contrast, to reduce DAI score in this DSS mice model (6.20 ± 1.30). Additionally, *L. lactis-*SlpB administration significantly limited the colon length shortening (*p* < 0.05) caused by the DSS administration, when compared with the DSS group (Figure 2C). It was more effective than *L. lactis* WT, which failed to do so (Figure 2C).

### Surface-Layer Protein B Protein Improves the Potential of *Lactococcus lactis* to Reduce Colon Mucosal Damage

**FIGURE 3:**
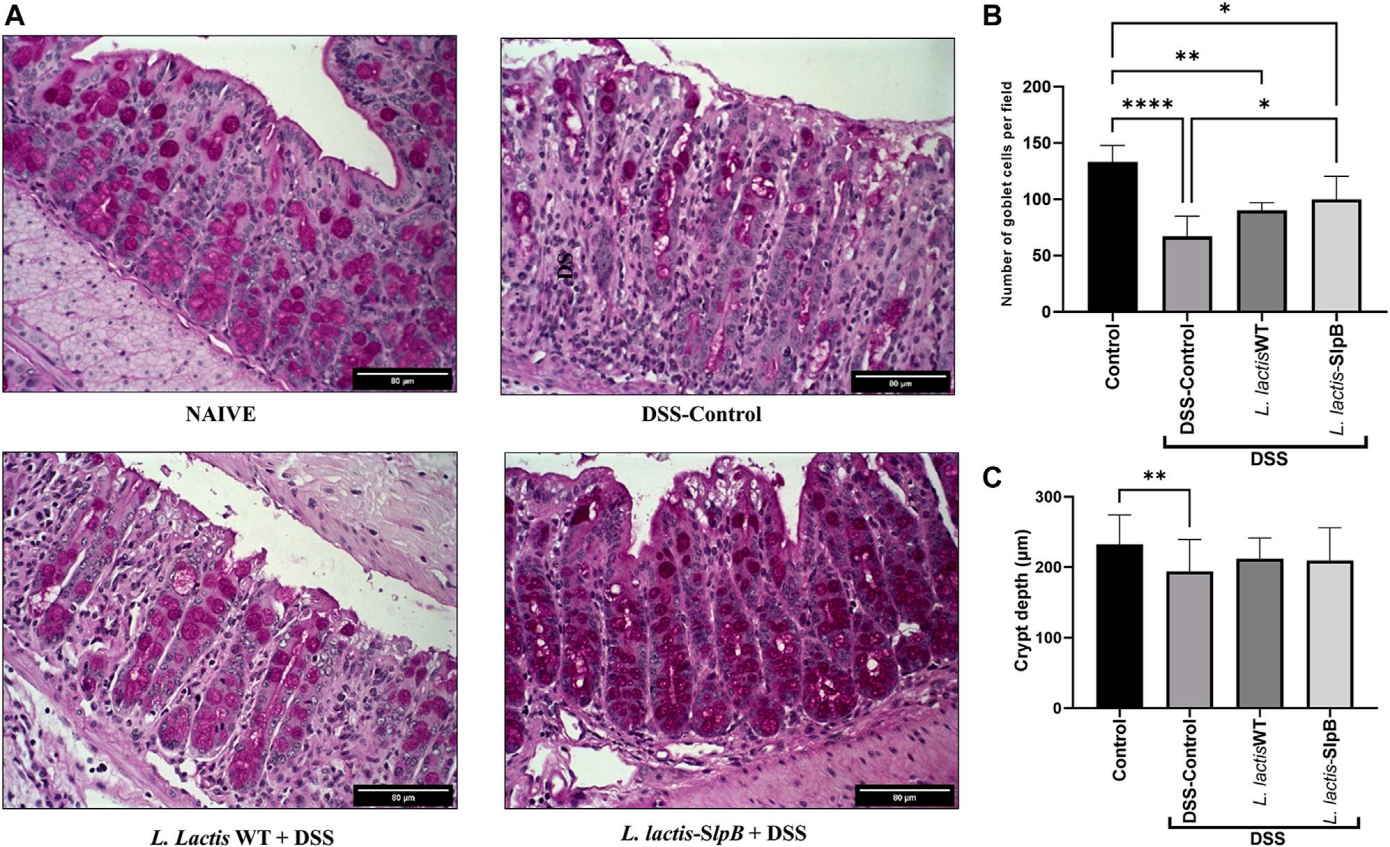
*L. lactis*-SlpB mitigates histological signs of DSS-colitis. Model images of micrographs for analysis of goblet cells in colon tissue **(A)** and the result of goblet cell quantification by field **(B)** and depth of colon intestinal crypts **(C)** are shown. The slides were stained in Periodic Acid-Schiff (PAS), goblet cells have intense purple-pink tones, and analyzed under ×40 magnification. The data represent the mean ± SD of 12 mice per group. One-way ANOVA and Tukey’s *post*-*hoc* tests were used for multiple comparisons. Asterisks represent statistically significant differences as follows: ∗*p* < 0.05, ∗∗*p* < 0.01, ∗∗∗*p* < 0.001, ∗∗∗∗*p* < 0.0001.

Histological analysis revealed that consumption of *L. lactis-*SlpB was able to mitigate the colon damage caused by DSS administration (Figure 2D). Precisely, it preserved colon morphological structure, reduced inflammatory cell infiltration in the lamina propria, submucosa, and muscular layer. Furthermore, animals that received *L. lactis* WT showed a slight decrease in mucosal damage, when compared with DSS control, but some ulcerations and a large infiltration of inflammatory cells were still observed. These results become evident through the analysis of the histopathological score, shown in Figure 2E, where, among the groups that consumed the DSS solution, the group *L. lactis-*SlpB showed significant differences in the histopathological score (3.14 ± 0.37), when compared with the group treated with *L. lactis* WT (4.00 ± 0.5, *p* < 0.05) and the DSS control group (5.0 ± 0.63, *p* < 0.0001). As expected, DSS-colitis induction resulted in a substantial decrease in goblet cells number in the DSS control group (67.08 ± 17.85 goblet cell/hpf). A significant increase in the number of goblet cells (Figures 3A, B) was exclusively observed in the group treated with *L. lactis-*SlpB (99.90 ± 20.51 goblet cell/hpf), when compared with the DSS control group (*p* < 0, 05). It was, however, not enough to re-establish the levels of the control group (133.1 ± 14.62 goblet cell/hpf, *p* < 0.05). In addition, *L. lactis* WT strain was not able to significantly increase the number of goblet cells (compared with the DSS-control group), and there was no statistical difference between *L. lactis-*SlpB and *L. lactis* WT strains (90.20 ± 6.64 goblet cell/hpf). A decrease in crypt depth (Figure 3C) was observed in the DSS Control group (191.1 μm ± 45.4, *p* < 0.05), compared with the Control group (232.3 μm ± 41.73). However, there was no statistical difference in the depth of the Crypts of Lieberkühn, between the treated groups *L. lactis* WT (212.5 μm ± 28.91) and *L. lactis-*SlpB (209.7 μm ± 46.30) with the control group (232.3 μm ± 41.73) and DSS group (191.1 μm ± 45.4).

### Wild-Type and Recombinant Strains Both Reduce Levels of Myeloperoxidase Activity and Eosinophilic Peroxidase

**FIGURE 4:**
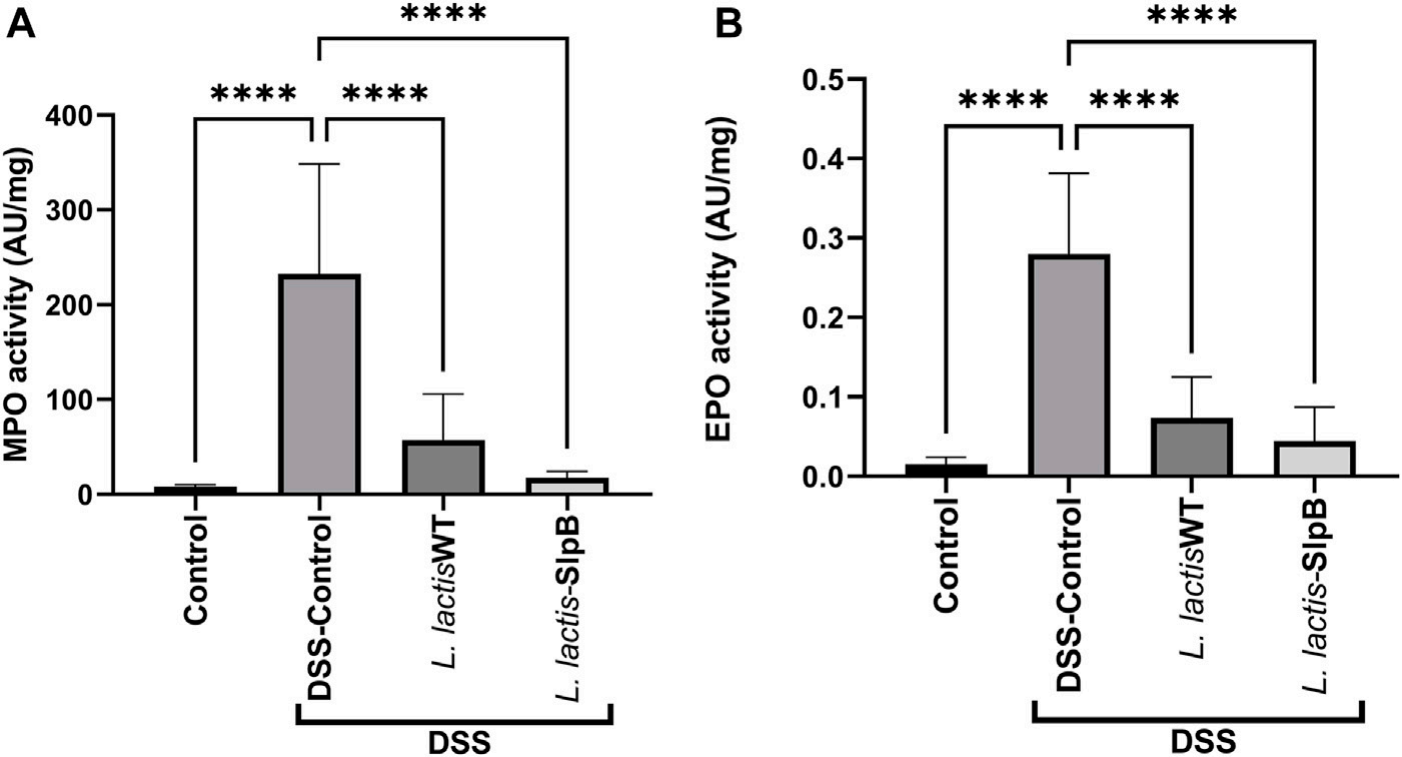
*L. lactis* Wild-type and *L. lactis*-SlpB strains prevent DSS-induced increase of myeloperoxidase (MPO) and eosinophilic peroxidase (EPO) activity. Quantification of the myeloperoxidase [MPO, **(A)**] and eosinophilic [EPO, **(B)**] enzymes in the colon tissue is shown. One-way ANOVA and Tukey’s *post*-*hoc* tests were used for multiple comparisons. The data represent the mean ± SD of six mice per group. Asterisks represent statistically significant differences as follows: ∗*p* < 0.05, ∗∗*p* < 0.01, ∗∗∗*p* < 0.001, ∗∗∗∗*p* < 0.0001.

Consumption of *L. lactis* WT and/or *L. lactis-*SlpB significantly decreased the amount of colon enzyme activity of MPO (57.27 ± 48.43 and 17.50 ± 6.65, respectively) (Figure 4A), with statistically significant differences for both treatments, when compared with the DSS control group (232.7 ± 115.9, *p* < 0.0001). The same scenario was repeated when the EPO enzyme activity was quantified (Figure 4B), where both strains, *L. lactis* WT and *L. lactis-*SlpB, proved effective to reduce EPO levels (0.07 ± 0.05 and 0.04 ± 0.04, respectively), with statistically significant differences, compared with the DSS Control group (0.28 ± 0.10, *p* < 0.0001). In addition, the results shown in Supplementary Figure S4 demonstrate high levels of secretory IgA (sIgA) in the inflamed control group DSS (91.30 μg/ml ± 40.93). However, no statistical differences between the groups *L. lactis* WT (63.54 μg/ml ± 17.17) and *L. lactis*-*slpB* (59.44 μg/ml ± 26.85 and control group (62.81 μg/ml ± 28.17) was observed.

### *Lactococcus lactis*-Surface-Layer Protein B Increases Expression of Genes Involved in Epithelial Barrier Protection

**FIGURE 5:**
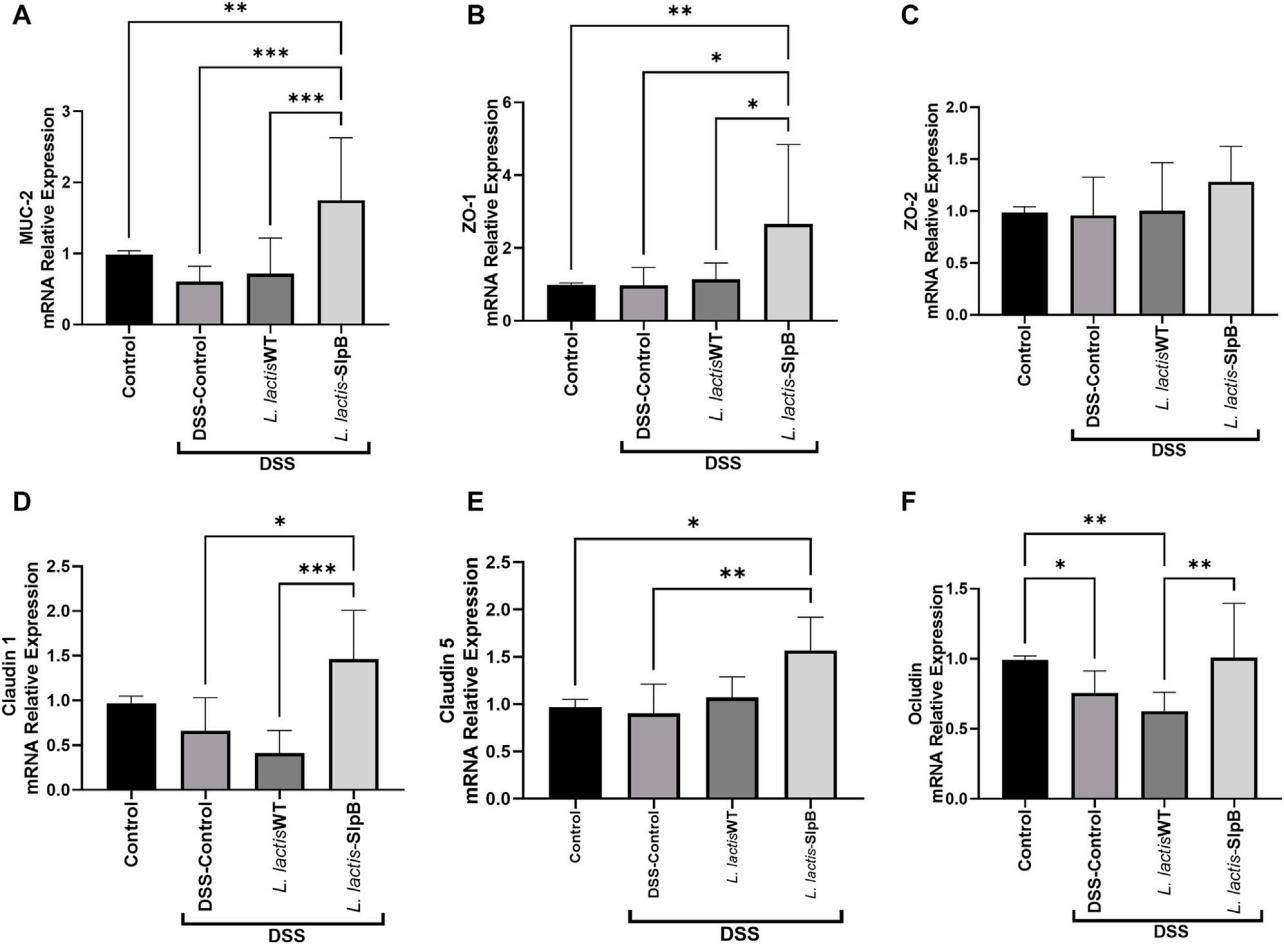
*L. lactis*-SlpB increases the expression of genes involved in epithelial barrier protection. Quantification of the expression of the genes Mucin 2 (*muc2*) **(A)**, Zonula Occludes 1 (*zo-1*) **(B)**, Zonula Occludes 2 (*zo-2*) **(C)**, Claudin-1 (*cln-1*) **(D)**, Claudin-5 (*cln-5*) **(E)**, and Occludin (*ocln*) **(F)**, in the mice colon, is shown. One-way ANOVA and Tukey’s *post*-*hoc* tests were used for multiple comparisons. The data represent the mean ± SD of six mice per group. Asterisks represent statistically significant differences as follows: ∗*p* < 0.05, ∗∗*p* < 0.01, ∗∗∗*p* < 0.001, ∗∗∗∗*p* < 0.0001.

In the context of DSS-induced colitis, consumption of *L. lactis*-*slpB* increased significantly (*p* < 0.001) the colonic mRNA expression levels (Figure 5) of *muc-2* gene and epithelial barrier genes *zo-1, cln-1,* and *cln-5* (1.75 ± 0.87; 2.65 ± 0.72; 1.46 ± 0.54; 1.56 ± 0.35, respectively), when compared with the DSS Control group (0.60 ± 0.21; 0.97 ± 0.49; 0.41 ± 0.25; 0.90 ± 0.31, respectively)*.* Interestingly, no difference in the expression levels of the *zo-2* gene was found between the experimental groups.

### Pro and Anti-Inflammatory Genes Implicated in Ulcerative Colitis are Modulated by the *Lactococcus lactis*-Surface-Layer Protein B Recombinant Strain

**FIGURE 6:**
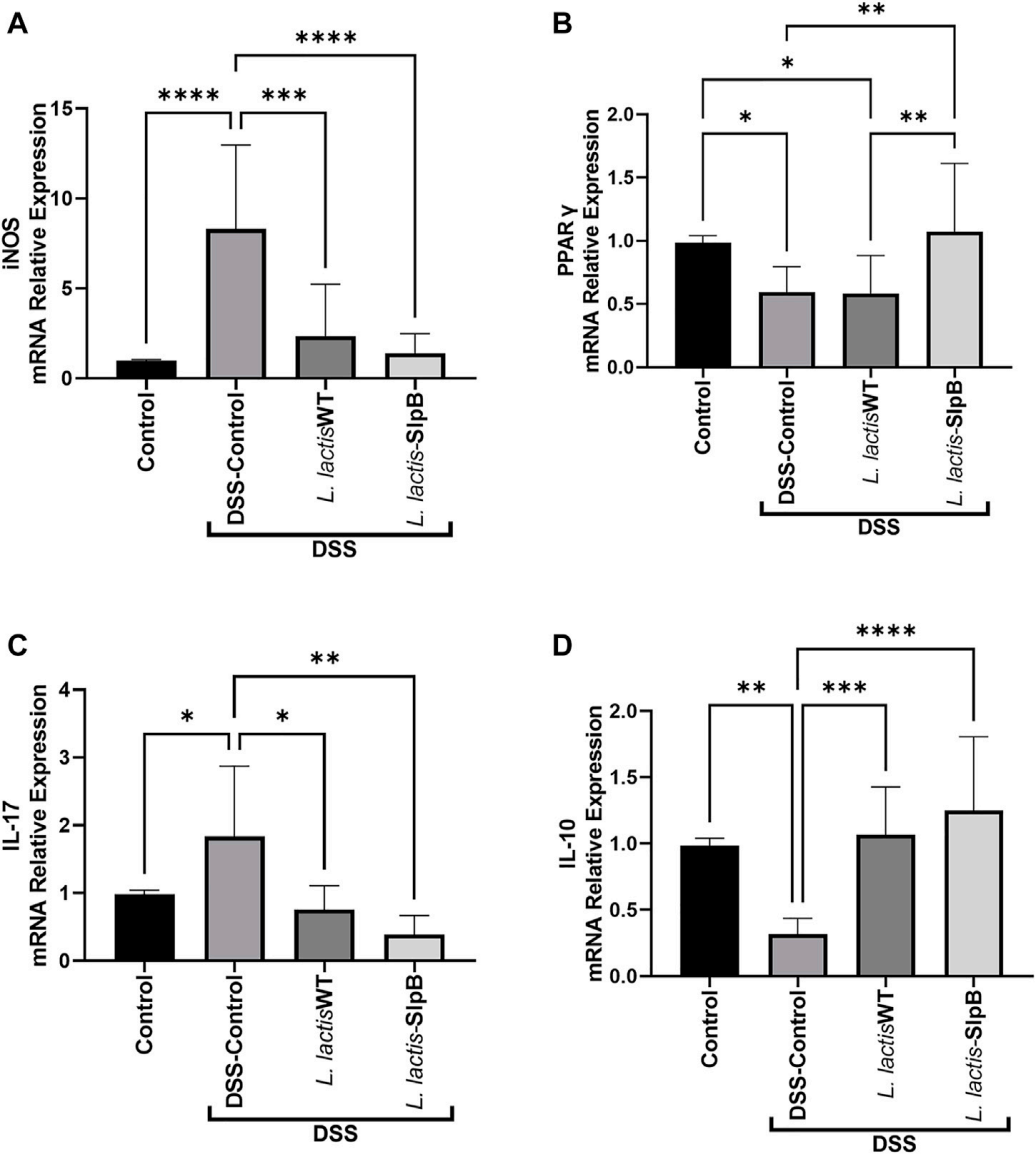
*L. lactis*-SlpB strain modulates expression of pro and anti-inflammatory genes implicated in ulcerative colitis. Quantification of the expression of the genes inducible nitric oxide synthase (*inos*) **(A)**, peroxisome proliferator-activated receptor-gamma (*pparg*) **(B)**, as well as interleukin-17 (*il-17*) **(C)** and interleukin-10 (*il-10*) **(D)** pro and anti-inflammatory cytokines, is shown. The data represent the mean ± SD of six mice per group. One-way ANOVA and Tukey’s *post*-*hoc* tests were used for multiple comparisons (*n* = 6). Asterisks represent statistically significant differences as follows: ∗*p* < 0.05, ∗∗*p* < 0.01, ∗∗∗*p* < 0.001, ∗∗∗∗*p* < 0.0001.

The increase in the *inos* gene expression levels triggered by DSS administration in the inflammatory control group (8.31 ± 4.66) were controlled by the administration of *L. lactis*-SlpB strain (1.39 ± 1.08, *p* < 0.01) (Figure 6A). On the other hand, mRNA levels of *pparγ* were decreased in the DSS Control group (0.59 ± 0.20) and in the *L. lactis* WT group (0.58 ± 0.30), but the *L. lactis*-SlpB administration restored significant levels of *pparγ* colonic expression (1.07 ± 0.54, *p* < 0.01) (Figure 6B). Regarding the expression of genes encoding pro and anti-inflammatory cytokines (Figures 6C, D), *L. lactis*-SlpB group showed significantly reduced levels of *il-17* gene expression (0.39 ± 0.27, *p* < 0.01), compared with the DSS control group (1.83 ± 1.03). Finally, the gene expression of anti-inflammatory cytokine *il-10* was reduced in the DSS Control group (0.31 ± 0.11), but was restored in the group that received the *L. lactis*-SlpB strain (1.25 ± 0.55, *p* < 0.001). However, there were no statistical differences in the colonic expression levels of *il-17* and *il-10* cytokines, between the groups receiving *L. lactis* WT or *L. lactis*-SlpB.

### *Lactococcus lactis-*Surface-Layer Protein B Strain Modulates Cytokine Production in the Mice Colon

**FIGURE 7:**
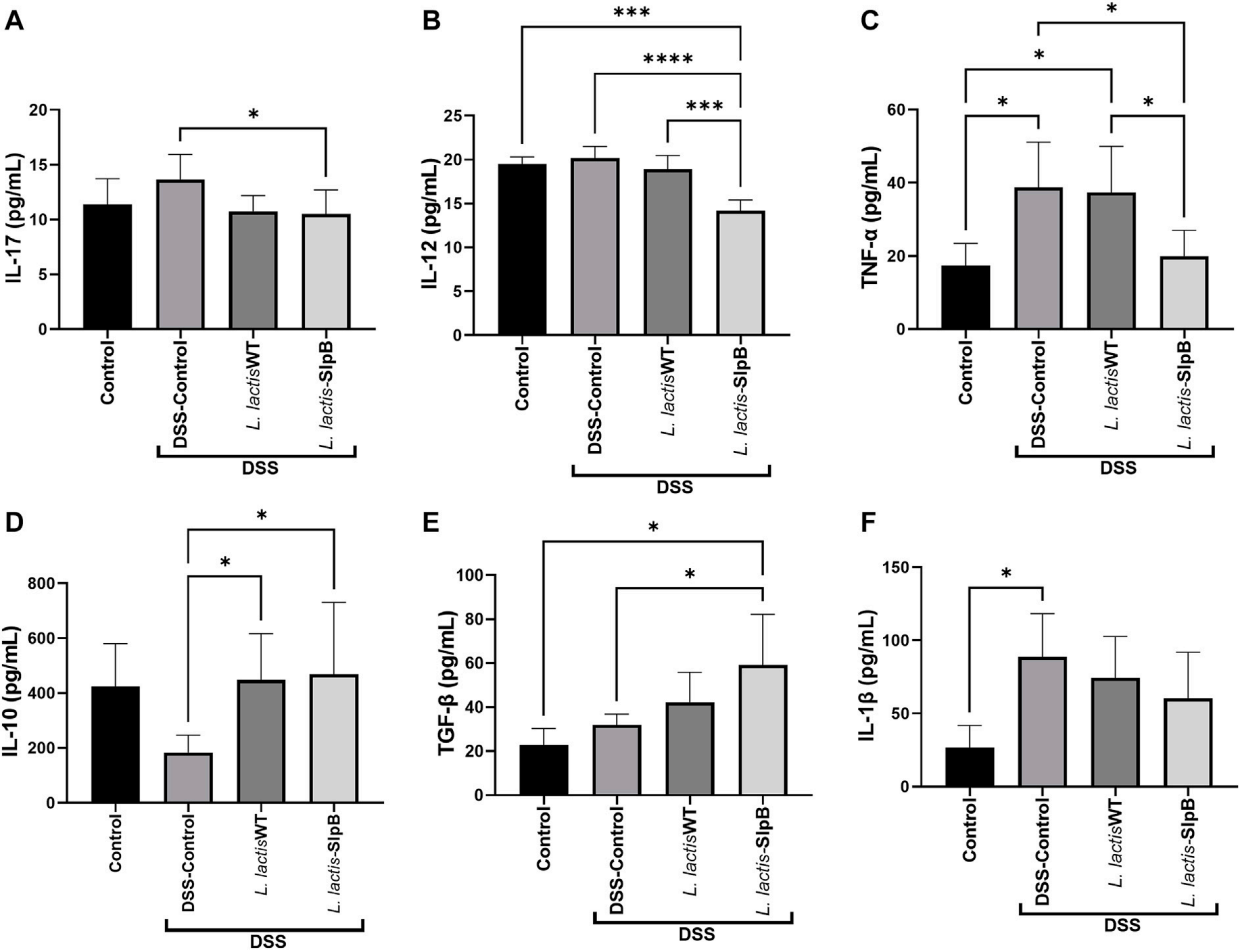
*L. lactis*-SlpB strain modulates cytokines production in the mice colon. Colonic cytokines concentrations levels of IL-17 **(A)**, IL-12 **(B)**, TNF-α, **(C)**, IL-10 **(D)**, TGF-β **(E)**, IL-1β **(F)** were quantified by enzyme-linked immunosorbent assay (ELISA). The data represent the mean ± SD of six mice per group. One-way ANOVA and Tukey’s *post*-*hoc* tests were used for multiple comparisons. Asterisks represent statistically significant differences as follows: ∗*p* < 0.05, ∗∗*p* < 0.01, ∗∗∗*p* < 0.001, ∗∗∗∗*p* < 0.0001.

Levels of colonic pro-inflammatory cytokines IL-17, IL-12, and TNF-α (13.66 ± 2.285, 20.17 ± 1.36; 17.41 ± 6.01, respectively) (Figures 7A–C) were increased in the DSS control group. In contrast, *L. lactis*-SlpB group showed significantly reduced levels of these cytokines, TNF-α, IL-17, and IL-12 (19.98 ± 7.04, *p* < 0.05; 10.51 ± 2.20, *p* < 0.05; 14.20 ± 1.20, *p* < 0.05, respectively), compared with the DSS control group. In addition, an increase in the colonic levels of IL-10 and TGF-β cytokines (Figures 7D, E) was observed in the group treated with *L. lactis*-SlpB (59.10 ± 23.14; 468.70 ± 261.60, *p* < 0.05, respectively) compared with the DSS Control group (31.96 ± 4.91; 183.80 ± 63.11, respectively). However, *L. lactis*-SlpB failed to decrease levels of Il-1β induced by DSS (Figure 7F).

## Discussion

Ulcerative colitis (UC) is an inflammatory bowel disease, which can be mimicked using *in vivo* models through induction with chemicals such as DSS (Wirtz et al., 2017). The inflammation occurring in UC affects the colonic epithelial cells and results in an impairment in the mucosal barrier function. In addition, colitis is marked by obvious clinical signs, such as weight loss, diarrhea, and occult blood in the feces (Zhang and Li, 2014). Some strains of lactic acid bacteria, such as *L. lactis* NCDO2118 (Luerce et al., 2014) and propionibacteria, such as *P. freudenreichii* CIRM-BIA 129 (Rabah et al., 2020) have already given promising results in alleviating the symptoms of UC. Precisely, *P. freudenreichii* 129 expresses a surface protein SlpB protein, which can be directly linked to its probiotic effects (Rabah et al., 2020). Thus, the DSS-induced mice model, the molecular tools for *L. lactis* NCDO 2118 to produce recombinant protein, and the SlpB protein from the *Pf* 129 strain, constitute the perfect scenario to test the potential of this protein to enhance the probiotic effects of other strains.

Several proteins expression models have already been successfully developed for *L. lactis* strains (Tavares et al., 2020). The xylose-induced model (XIES), developed exclusively for *L. lactis* NCDO 2118 by Miyoshi et al. (2004), not only expresses but also uses the mechanism of secretion and targeting of the protein to the extracellular medium, allowing the correct targeting of the SlpB surface protein. Besides, due to simple metabolism and rapid growth (12–24 h), in contrast to *P. freuderinchii* 2–3 days to reach the stationary growth phase, *L. lactis* began to be used for the production of recombinant proteins in the cytoplasm or secreted into the extracellular medium (Carvalho et al., 2017).

*L. lactis* NCDO 2118 and *P. freudenreichii* CIRM-BIA 129, as well as the action of some surface proteins in a purified way, can bring relief in weight loss in mice with colitis or other models of inflammation (Luerce et al., 2014; Cai et al., 2018; Do Carmo et al., 2020; Rabah et al., 2020). Luerce et al. (2014) showed that the *L. lactis* NCDO2118 strain can prevent colon shortening in the context of colitis. In our work, mitigation of inflammation was enhanced by the presence of SlpB. Indeed, concerning colon length, the wild-type strain alone did not show efficacy in this DSS mice model. Furthermore, although *L. lactis* NCDO2118 WT gave good results by decreasing the histopathological score, in accordance with Luerce et al. (2014), our results indicate that the presence of the SlpB protein further enhanced the histopathological score, when compared with the *L. lactis* NCDO 2118 group. The preservation of goblet cells is an important aspect of probiotic mechanisms of action. These cells produce mucus, which serves as a barrier preventing the direct adhesion of microorganisms to the epithelium (Abrantes et al., 2020). It is worth noting that the *L. lactis*-SlpB strain increased the expression of the *muc*-2 gene and restored goblet cells in animals treated with DSS. The goblet cells are responsible for producing the mucus that covers the intestinal mucosa, and high levels of sIgA can be found in the mucus layer of the intestine in healthy individuals (Rogier et al., 2014). However, increased IgA secretion may be related to an inflammatory response caused by disturbances in the ileum intestinal barrier, as shown by Rabah et al. (2020). In the present work, only the DSS group exhibited high levels of sIgA, but no significant differences between groups were found. Moreover, preservation of the epithelium, demonstrated in the histology of animals that received *L. lactis*-SlpB, is consistent with the increased expression of the *zo-1*, *cld-1*, *cld-5*, and *ocln* genes responsible for the expression of tight junction proteins, maintaining the epithelial barrier function and controlling cell permeability (Landy et al., 2016). It is plausible that the SlpB protein plays a central role in reinforcing the epithelial barrier, but further studies are needed, such as treatment with purified SlpB protein and monitoring of intestinal permeability, to conclude this statement.

Regarding the inflammatory cells infiltrate, it was visibly attenuated in the group treated with *L. lactis* NCDO2118 and even smaller in the animals treated with *L. lactis*-SlpB. This infiltrate is composed of mononuclear and polymorphonuclear cells, and in ulcerative colitis, increased levels of neutrophils and eosinophils are mainly observed (Villanacci et al., 2013). Therefore, we quantified the colonic activity of myeloperoxidase (MPO) and eosinophilic peroxidase (EPO), as a means of indirect determination of neutrophils and eosinophils, respectively, in the colon of animals. The results obtained in the quantification of MPO and EPO enzymes in this work corroborate those described by Han et al. (2021), where the DSS group enhanced activity of both enzymes in the colon.

PPARγ is a regulator of intestinal inflammation. It inhibits transcription of pro-inflammatory cytokine genes, such as *ifn-γ*, and the inducible nitric oxide synthase (*inos*) gene (Dubuquoy et al., 2006; Rabah et al., 2020). The activation of the *inos* expression is responsible for mediating the accumulation of nitric oxide that results in oxidative stress and it is directly linked to gastrointestinal immunopathology, such as ulcerative colitis (Kolios et al., 2004). We observed that the DSS-induced colitis resulted in a significant increase in the expression of nitric oxide synthase corroborating the results obtained in the work of Rabah et al. (2020). However, *L. lactis*-SlpB triggered an increase in the expression of the *ppar*γ gene, showing an effect not found with the administration of the *L. lactis* NCDO 2118 wild-type strain. Patients with ulcerative colitis have impaired expression of *ppar*γ in the colon and the increased expression of this gene can lead to the inhibition of inflammatory cytokines such as IL-1β and TNF-α (Dubuquoy et al., 2006). We accordingly observed a reduction in the levels of TNFα in the animals treated with the *L. lactis*-SlpB, where the *L. lactis* NCDO 2118 wild-type strain failed to prevent the increase in cytokine secretion caused by DSS-induced colitis.

Immune response, i.e., pro and anti-inflammatory cytokines, is one of the main mediators of the pathogenesis of colitis (Ko and Auyeung, 2014). In this aspect, bacterial surface proteins may moderate dysregulation of cytokines, as demonstrated by the effects of the SlpA protein from *Lactobacillus acidophilus* CICC in the DSS-induced colitis model (Cai et al., 2018). The impact of the SlpB protein on the IL-12 cytokine expression has already been demonstrated by Do Carmo et al. (2020). Indeed, consumption of the wild strain of *Pf* 129 triggered a decrease in this cytokine during 5-FU-induced mucositis, but the same was not observed as a result of the consumption of the knockout strain for the *slpB* gene (*Pf* 129Δs*lpB*). Furthermore, activation of IL-10 and TGF-β secretion by the *L. lactis*-SlpB strain was also observed here, an important factor that contributes to the attenuation of the inflammatory response in the colon caused by DSS. The anti-inflammatory cytokine IL-10 can inhibit the production of IL-1β, IL-6, and TNF-α. However, to access IL-10 protective effect against colitis, IL-10 signaling pathway must be triggered before the induction of DSS colitis (Li and He, 2004). The mechanism of action of the SlpB protein also appears to be aimed at limiting the inflammatory process, mainly containing IL-17, *via* regulation of Th17 cells. Colliou et al. (2017) demonstrated that propionibacteria that enrich the microbiota of infants through breastfeeding can attenuate the incidence of necrotizing enterocolitis through the regulation of Th17 cells.

## Conclusion

S-layer proteins have a great potential to mediate host–probiotic interactions *via* intestinal cells, which are important to maintain gut immunity homeostasis and to mitigate inflammatory diseases. Mice that received *L. lactis*-slpB showed a significant reduction in colitis severity symptoms. Thus, it is plausible that the presence of SlpB protein in the *L. lactis* NCDO 2118 strain increased its potential to control the effects and symptoms of DSS-induced colitis, such as decreased DAI. Further studies involving purified SlpB protein and its expression in other organisms are needed to unravel its ability to enhance probiotic effects. This work demonstrates that *L. lactis* NCDO 2118 harboring SlpB recombinant protein prevents the inflammatory process during DSS-induced colitis in mice, opening perspectives for the development of new probiotic functional foods for personalized nutrition in the context of IBD.

## Data Availability

The raw data supporting the conclusion of this article will be made available by the authors, without undue reservation.

## Supplementary Material

SUPPLEMENTARY FIGURE S1 Experimental design to explore anti-inflammatory effects of L. lactis-SlpB in DSS-colitis mice model. C57BL6 mice were divided into 4 groups and receiving or not different treatments for 7 days simultaneous colitis induction by 1.7% DSS in drinking water for 7 days prior to euthanasia. Different disease parameters were monitored to study the severity of colitis.

SUPPLEMENTARY FIGURE S2 Western Blotting detection of surface layer protein SlpB. PVDF membranes were treated using rabbit antibodies raised against P. freudenreichii 129 surface layer protein SlpB. **A-** Positive control - surface proteins (including SpB) from the Pf 129 strain extracted by Guanidine Chloride (20 ug of proteins). L. lactis-SlpB non-induced (Growth with Glucose-plasmid repressor): **B-** Total protein (10 μg protein); **C-** Supernatant protein (10 ug of proteins), D- Surface proteins (Guanidine Chloride Extract - 50 ug of proteins L. lactis-SlpB induced by Xylose: **E-** Total protein (10 μg protein); **F-** Supernatant protein (10 ug of proteins), **G-** Surface proteins (Guanidine Chloride Extract - 50 ug of proteins).

SUPPLEMENTARY FIGURE S3 (A) Liquid intake (ml/mice) and (B) Food consumption (g/mice) during experimental procedure of DSS colitis induction. Results were expressed as means ± standard deviations (SD). No significant differences were found.

SUPPLEMENTARY FIGURE S4 L. lactis Wild-type and L. lactis-SlpB strains did not alter the secretory IgA production. The concentration of secretory IgA in the small intestine. The data represent the mean ± SD of 12 mice per group. The One-Way ANOVA and Tukey post-hoc tests were used for the multiple comparisons. No significant difference was found.
