# Supplementary figures and images for: SlpB Protein Enhances the Probiotic Potential of L. lactis NCDO 2118 in Colitis Mice Model
Source: Front Pharmacol. 2021 Dec 20;12:755825. doi: 10.3389/fphar.2021.755825 (PMC8721164; doi:10.3389/fphar.2021.755825)

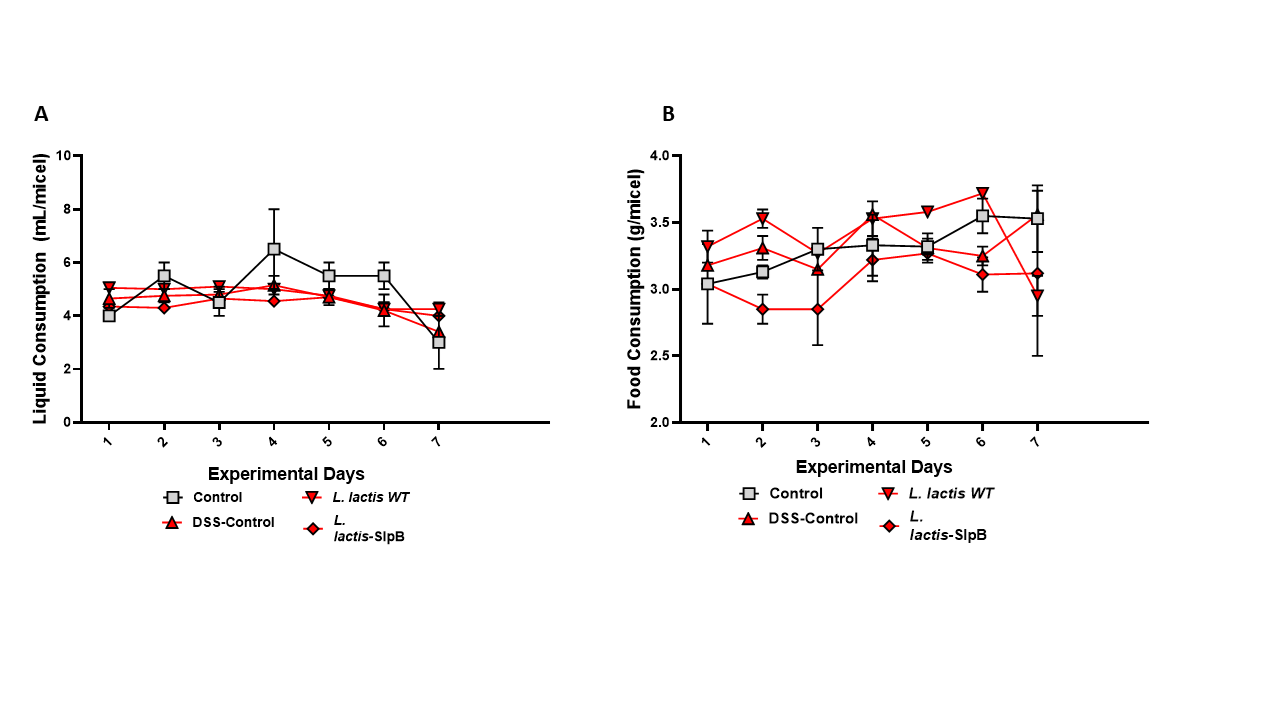

Supplement: Supplementary file 1 [file Image3.tif]

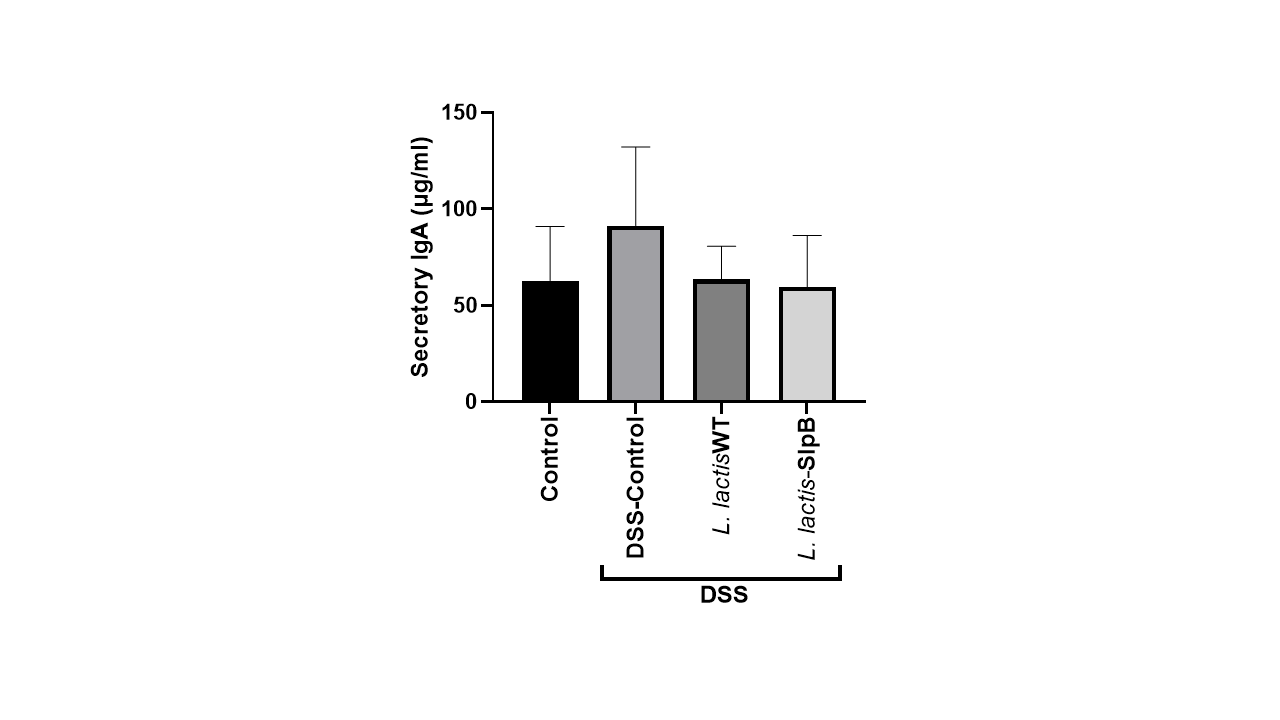

Supplement: Supplementary file 2 [file Image4.tif]

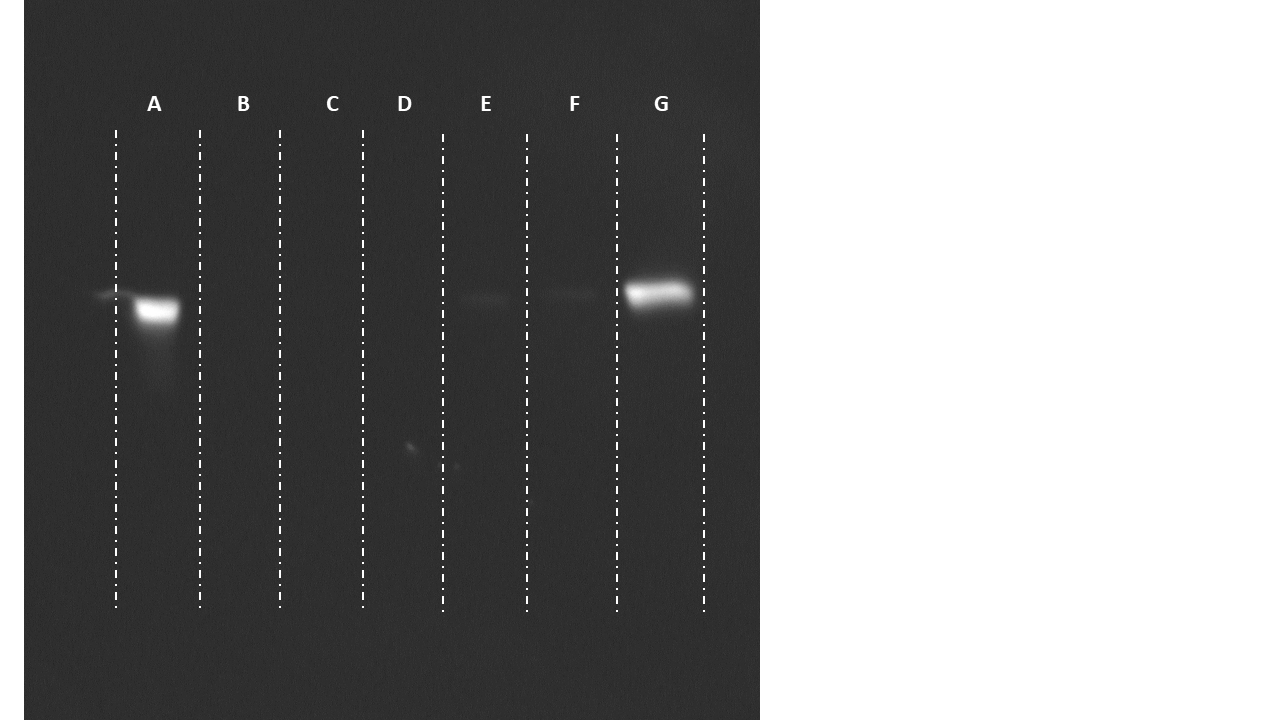

Supplement: Supplementary file 3 [file Image2.tif]

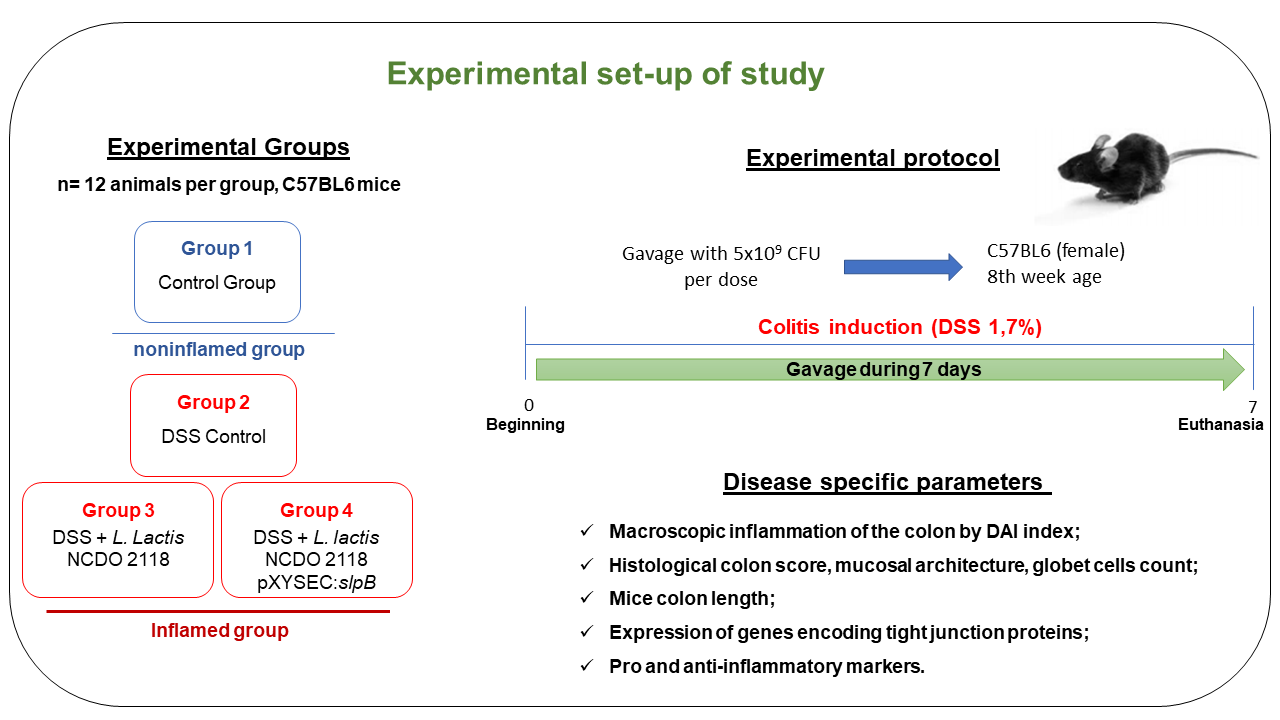

Supplement: Supplementary file 4 [file Image1.tif]
